# Left Atrial Function Predicts Atrial Arrhythmia Recurrence Following Ablation of Long-Standing Persistent Atrial Fibrillation

**DOI:** 10.1161/CIRCIMAGING.123.015352

**Published:** 2023-06-08

**Authors:** Habib Rehman Khan, Haci Yakup Yakupoglu, Ines Kralj-Hans, Shouvik Haldar, Toufan Bahrami, Jonathan Clague, Anthony De Souza, Wajid Hussain, Julian Jarman, David Gareth Jones, Tushar Salukhe, Vias Markides, Dhiraj Gupta, Rajdeep Khattar, Tom Wong

**Affiliations:** London Health Sciences Centre, University of Western Ontario, London, Canada (H.R.K.).; Cardiology Department, Royal Brompton and Harefield Hospitals, Guy’s and St Thomas’ NHS Trust, London, United Kingdom (H.Y.Y., I.K.-H., S.H., T.B., J.C., A.D.S., W.H., J.J., D.G.J., T.S., V.M., R.K.).; National Heart and Lung Institute, Imperial College London, United Kingdom (R.K., T.W.).; Liverpool Heart and Chest Hospital, United Kingdom (D.G.).

**Keywords:** ablation, atrial fibrillation, left atrial function, left atrial strain, sinus rhythm

## Abstract

Left atrial (LA) function following catheter or surgical ablation of de-novo long-standing persistent atrial fibrillation (AF) and its impact on AF recurrence was studied in patients participating in the CASA-AF trial (Catheter Ablation vs. Thoracoscopic Surgical Ablation in Long Standing Persistent Atrial Fibrillation).

**Methods:**

All patients underwent echocardiography preablation, 3 and 12 months post-ablation. LA structure and function were assessed by 2-dimensional volume and speckle tracking strain measurements of LA reservoir, conduit, and contractile strain. Left ventricular diastolic function was measured using transmitral Doppler filling velocities and myocardial tissue Doppler velocities to derive the e’, E/e’, and E/A ratios. Continuous rhythm monitoring was achieved using an implantable loop recorder.

**Results:**

Eighty-three patients had echocardiographic data suitable for analysis. Their mean age was 63.6±9.7 years, 73.5% were male, had AF for 22.8±11.6 months, and had a mean LA maximum volume of 48.8±13.8 mL/m^2^. Thirty patients maintained sinus rhythm, and 53 developed AF recurrence. Ablation led to similar reductions in LA volumes at follow-up in both rhythm groups. However, higher LA emptying fraction (36.3±10.6% versus 27.9±9.9%; *P*<0.001), reservoir strain (22.6±8.5% versus 16.7±5.7%; *P*=0.001), and contractile strain (9.2±3.4% versus 5.6±2.5%; *P*<0.001) were noted in the sinus rhythm compared with AF recurrence group following ablation at 3 months. Diastolic function was better in the sinus rhythm compared with the AF recurrence group with an E/A ratio of 1.5±0.5 versus 2.2±1.2 (*P*<0.001) and left ventricular E/e’ ratio of 8.0±2.1 versus 10.3±4.1 (*P*<0.001), respectively. LA contractile strain at 3 months was the only independent predictor of AF recurrence.

**Conclusions:**

Following ablation for long-standing persistent AF, improvement in LA function was greater in those who maintained sinus rhythm. LA contractile strain at 3 months was the most important determinant of AF recurrence following ablation.

**Registration:**

URL: https://www.clinicaltrials.gov; Unique identifier: NCT02755688

**Clinical Perspective**

Cardiac parameters such as increased left atrial (LA) size and reduced reservoir function in paroxysmal atrial fibrillation (AF), early persistent, and persistent AF are associated with AF recurrence following ablation. There is sparse information on the impact of ablation on LA size and function in patients suffering from long-standing persistent AF. The recently reported CASA-AF randomized-controlled trial (Catheter Ablation vs. Thoracoscopic Surgical Ablation in Long Standing Persistent Atrial Fibrillation) showed similar success rates between catheter ablation and thoracoscopic surgical ablation in patients with long-standing persistent AF. This substudy from the CASA-AF trial shows LA and overall cardiac function does not differ between catheter and surgical ablation. However, irrespective of the ablation technique, patients with lower LA contractile function following ablation are more likely to have AF recurrence. This study suggests that following ablation for long-standing persistent AF, those with markedly reduced LA contractile function are at a higher risk of developing AF recurrence and require closer monitoring during follow-up, focusing on alternative rate and rhythm control strategies. This study provides background information on future research to study the relationship of LA strain with fibrotic changes on cardiac magnetic resonance imaging or histology.

Atrial fibrillation (AF) is an epidemic with the highest prevalence in industrialized countries, associated with an increased risk of cardiovascular complications and death.^1^ AF is associated with poor left atrial (LA) function and adverse remodeling manifest by chamber dilatation.^2^ The duration of AF is positively correlated with LA dilatation and worsening LA function.^3–7^ Metabolic and neurohormonal changes lead to advanced electro-anatomic LA remodeling causing irreversible changes such as fibrosis^6,8^ resulting in postablation mechanical stunning and early AF recurrence following ablation.^9^ The changes in LA volume and function also reflect left ventricular (LV) diastolic dysfunction, which is difficult to reliably quantify during AF. Parameters such as E/e’ and LA strain are more sensitive in discriminating left ventricle diastolic dysfunction from normal function and may help to monitor functional changes once sinus rhythm (SR) is restored.^10–12^ Left ventricle diastolic dysfunction algorithms in recent guidelines do not accurately reflect LA pressure directly, and studies show a better correlation with LA strain data.^13,14^

The LA functions sequentially as a reservoir, conduit, and pump during the cardiac cycle.^15^ Reservoir function is the elastic ability of the LA to stretch during filling. Conduit function is the passive recoil of the LA walls during early emptying of blood from the LA into the LV from the time of mitral valve opening to the onset of atrial contraction in SR or to the end of diastole in AF. Contractile function (pump) is the late active emptying of blood into the LV from the onset of atrial contraction to end-diastole in patients with SR and is absent in AF.

Transthoracic echocardiography comprehensively assesses LA structure and function by deriving LA volumes and using 2D speckle tracking strain imaging to quantify myocardial deformation during LA relaxation and contraction.^15^ Timely evaluation of LA function may help identify patients who are likely to maintain SR post-ablation.^16,17^ Several studies have used volume and 2D strain measurements to investigate changes in LA anatomy and function in patients with AF, but involved mainly paroxysmal or early persistent AF rather than long-standing persistent AF (LSPAF, lasting more than 12 months).^4,16,18^

In the CASA-AF trial (Catheter Ablation vs. Thoracoscopic Surgical Ablation in Long Standing Persistent Atrial Fibrillation), we previously showed similar efficacy of catheter ablation and surgical ablation for maintaining SR in patients with LSPAF.^19^ In this substudy, we hypothesized that LA function is better in patients who maintain SR following ablation compared to those who have AF recurrence and may be an important predictor of AF recurrence.

The aims of this sub-study were (i) to assess the effect of ablation therapy on LA structure and function in LSPAF; (ii) to compare LA structure and function in patients maintaining SR and those with AF recurrence following ablation, and (iii) to determine the independent predictors of AF recurrence following ablation.

## Methods

The data that support the findings of this study are available from the corresponding author upon reasonable request.

### Study Population

Patients with LSPAF enrolled into the CASA-AF randomized-controlled trial (Catheter Ablation vs. Thoracoscopic Surgical Ablation in Long Standing Persistent Atrial Fibrillation) who underwent catheter or surgical ablation were followed up for 12 months. The protocol of the CASA-AF trial and the primary outcome analysis were published previously.^19,20^ The study was approved by the National Health Research Authority (15/SC/0023), and all participants gave written consent. Included patients were >18 years old with long-standing persistent AF (>12 months’ duration), European Heart Rhythm Association symptom score >2, left ventricular ejection fraction ≥40%, suitable for either ablation procedure. Those with more than mild valvular heart disease, contra-indication to anticoagulation, a cerebrovascular accident within the previous 6 months, previous cardiothoracic surgery, prior AF ablation, or other severe concomitant conditions were excluded. AF recurrence at follow-up was determined by continuous heart rhythm monitoring enabled by an internal loop recorder (Reveal LINQ, Medtronic, MN). AF recurrence was defined as >30 seconds of AF occurring after the blanking period of 3 months. The 3-month blanking period was recommended in international AF management guidelines to allow the ablation lesion to heal and for the rhythm to stabilize. AF recurrence during this early period has not been associated with clinical outcomes at 1 year.^21,22^

As part of the study protocol, echocardiographic measurements of the LA parameters were performed at baseline, 3 months, and 12 months after ablation. Only patients in SR during the echocardiographic examination at follow-up were included in the study to allow all aspects of LA function, including contractile, to be evaluated.

Patients with thyroid diseases were defined as those with metabolically active disease requiring treatment. Chronic kidney disease was based on an eGFR <60 mL/minute. Coronary artery disease was defined as previous revascularization or coronary angiography showing >70% stenosis in a major epicardial coronary artery. Respiratory disease was defined as chronic airways disease on regular treatment. A positive history of alcohol consumption was defined as >2 units of alcohol consumption per week.

### Echocardiography

**Figure 1.**
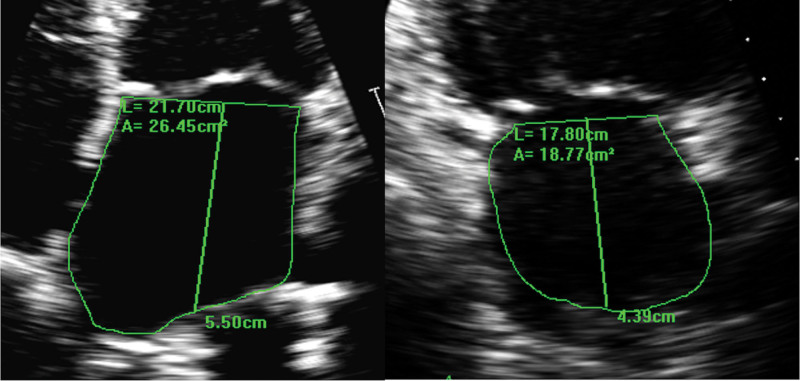
**Left atrial (LA) volume measurement using the biplane area-length method.** Zoomed view of the LA in the apical 4-chamber view is on the left and apical 2-chamber view on the right. A indicates area; and L, length.

Transthoracic echocardiography was performed using Philips IE33 (Andover, MA) and GE Vivid E9/ E95 machines (Chicago, IL). A standard minimum dataset of images was acquired as per guidelines^23^ with a minimum frame rate of 55 Hertz and 3 cardiac cycles. LA volumes were calculated using the biplane area-length method (Figure 1) and included the maximum (LAmax: volume before mitral valve opening), minimum (LAmin: volume at the beginning of mitral valve closure), and volume at the onset of the P-wave (LAp-wave). All measurements were indexed to body surface area. The LA emptying fraction (LAEF, %) was defined as the LAmax minus the LAmin divided by the LAmax. The LA sphericity index (%) was defined as the maximum transverse diameter divided by the maximum anteroposterior length. LA reverse remodeling was defined as the percentage reduction in LAmax after ablation compared with baseline. Delta LAmax was defined as the absolute volume change in LAmax volume from baseline to 3 months follow-up. Delta LAEF was the absolute change in the LAEF from baseline to 3-month follow-up. The echocardiographic measurements for each LA volume dataset and derivations are shown in Table S1. Left ventricle diastolic dysfunction was assessed using transmitral Doppler E and A filling velocities and myocardial tissue Doppler e’ velocities. At baseline during AF, it was only possible to measure E/e’, whereas in SR, both trans-mitral Doppler E/A ratio and LV E/e’ were used to measure left ventricle diastolic dysfunction.

**Figure 2.**
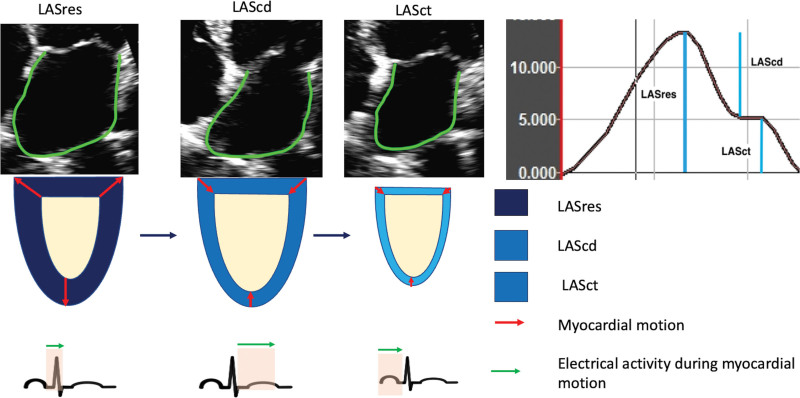
**Two-dimensional left atrial strain data.** The diagram shows reservoir, conduit, and contractile function measurements of left atrium (LA) in a patient in sinus rhythm. cd indicates conduit function; ct, contractile function; LAS, left atrial strain; and res, reservoir function.

LA strain measurements were made offline by one experienced operator,^24^ using vendor-independent software (TomTec imaging systems, version 1.4, Unterschleissheim, Germany), and averages reported for both 4-chamber and 2-chamber views using the onset of QRS as a zero reference for cardiac cycle. Strain measurements reflected the peak % change in length of the LA myocardium during the 3 phases of the cardiac cycle (Figure 2). LA strain measurements were taken during the reservoir, conduit and contractile phases of LA function denoted as left atrial strain reservoir function (LASres), LAScd, and left atrial strain contractile function (LASct), respectively. Delta LASres was defined as the absolute change in the LA LASres from baseline to 3 months follow-up.

Inter-observer variability of LAmax volume, LASres, and LASct measurements was assessed by 2 independent expert evaluators on a random sample of 48 patients. Interclass correlation coefficients were 0.72, 0.86, and 0.78, respectively.

### Statistical Analysis

Statistical analysis was performed using SPSS version 25. Data were compared for differences in demographic and echocardiographic parameters between ablation modalities and rhythm outcomes. All variables were normally distributed and analyzed using a 2-way ANOVA and shown as mean±SD and 95% CI. Comparisons between the 2 rhythm groups were performed using ANCOVA to adjust for baseline differences. Categorical values are expressed as percentages and analyzed using the χ^2^ test. Cox regression analysis was performed to examine factors associated with time to first AF recurrence following the 3 months blanking period. Patients without AF recurrence were censored at the point of the last follow-up. Univariable analysis was performed for each parameter. A multivariable Cox regression analysis was performed on variables with a *P*<0.1. Collinearity between variables was tested by using the variance inflation factor, and variables with high collinearity (variance inflation factor >10) were eliminated from the Cox regression analysis. LAEF, delta LASres, and LASres had high variance inflation factor and, therefore, delta LASres was excluded from the prediction model. Two separate multivariate analyses were performed using first, LA measurements at baseline and then those made after the 3-month blanking period to identify independent predictors of AF recurrence. Receiver operating characteristic curves were constructed for each of the independent predictors to identify the optimal cutoff value for predicting AF recurrence. Kaplan-Meier survival curve analysis was then performed using the cutoff value to show freedom from AF during follow-up and estimate the diagnostic accuracy. A *P*<0.05 was considered statistically significant.

## Results

Of the 115 CASA-AF participants, 32 patients were excluded, of whom 7 had inadequate image quality, 23 had incomplete data on baseline or serial echocardiography, and 2 patients were in AF requiring repeat ablation. Baseline characteristics were not affected by these exclusions (Table S2). Among the remaining 83 patients, mean age was 63.6 (±9.7) years, 73% were male, 52% had hypertension, 7% had diabetes, 13% had coronary artery disease, 7% were active smokers, 4% had a previous stroke or transient ischemic attack, 6% had chronic kidney disease, 6% had thyroid disease, and 16% had respiratory disease (Table 1). Forty-six patients had catheter and 37 had thoracoscopic surgical ablation in 83 patients. There were no significant differences in the baseline clinical characteristics or AF recurrence rates between the 2 ablation strategies (Table S3). Among the 83 patients, 53 (64%) developed paroxysmal AF recurrence after the 3-month blanking period. Seventy-eight and 81 patients were in SR during the 3 and 12 months echocardiograms, respectively.

**Table 1.**
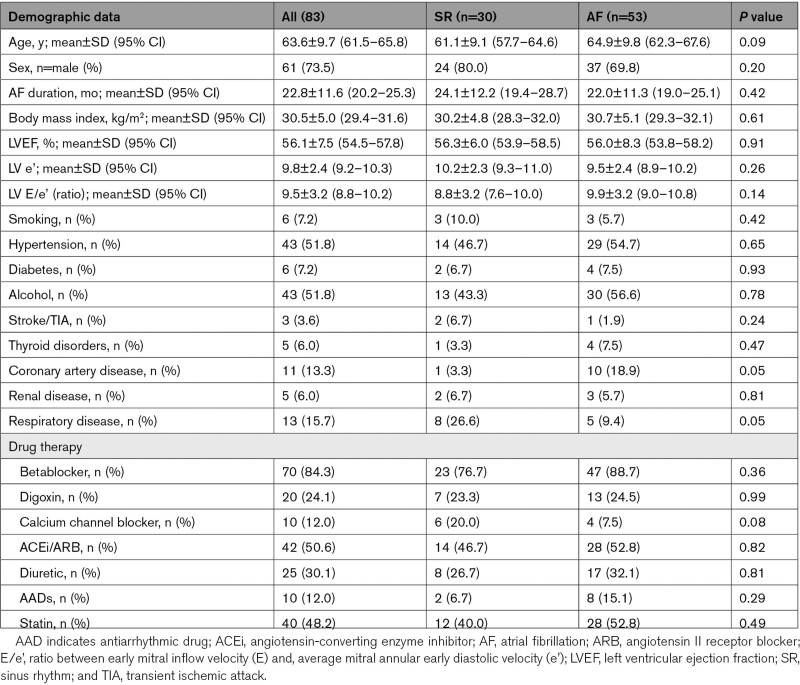
Comparison of Baseline Characteristics of the Sinus Rhythm Versus Atrial Fibrillation Recurrence Groups

### LA Volumes, Strain Data, and LV Diastolic Function in the Total Sample

**Figure 3.**
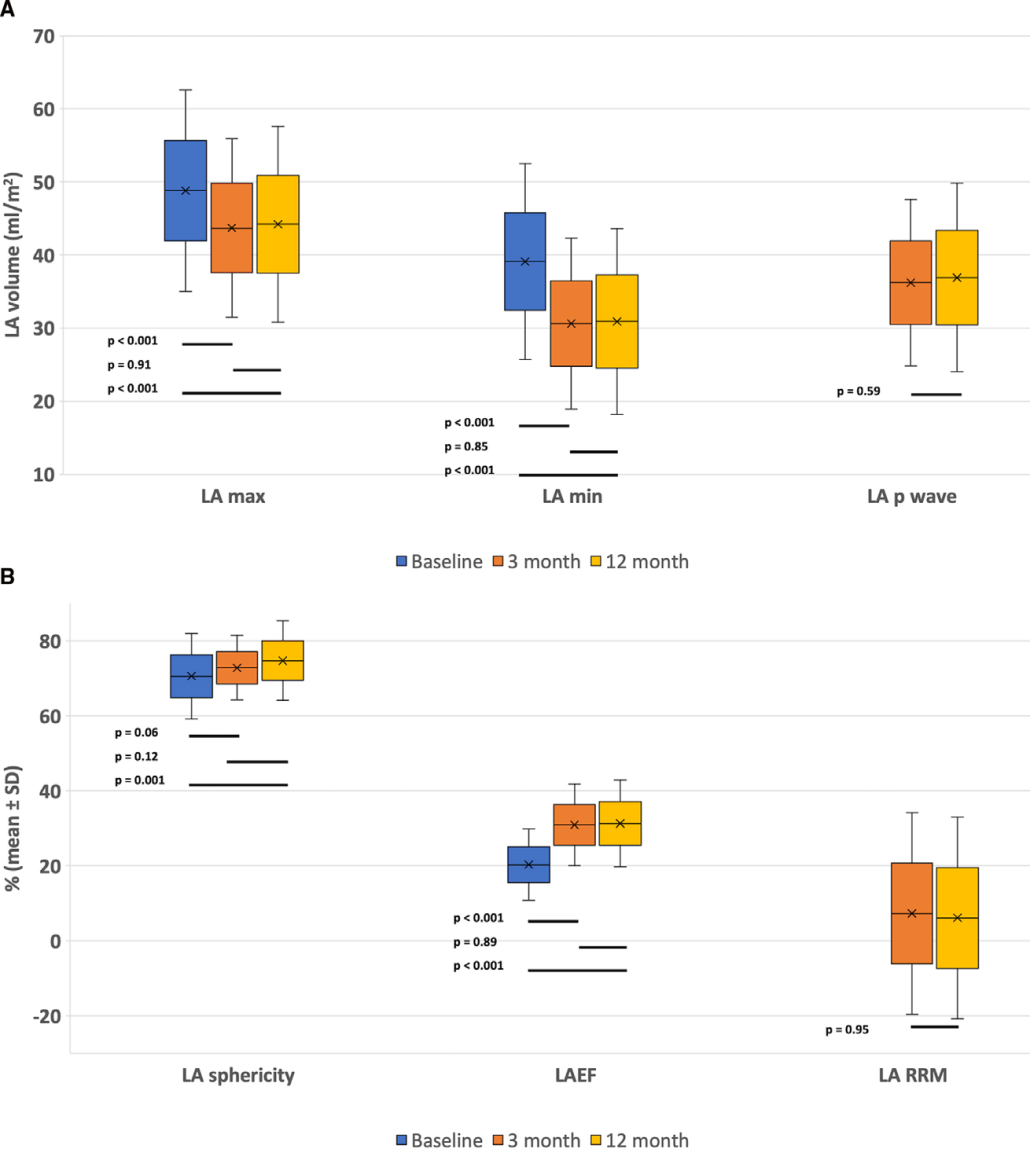
**Changes in left atrial (LA) parameters in the total sample. A**, Changes in LA maximum, minimum, and p wave volumes from baseline to 12 months in the total study population. **B**, Changes in LA ejection fraction (LAEF), sphericity index, and LA reverse remodelling (LA-RRM) from baseline to 12 months.

**Figure 4.**
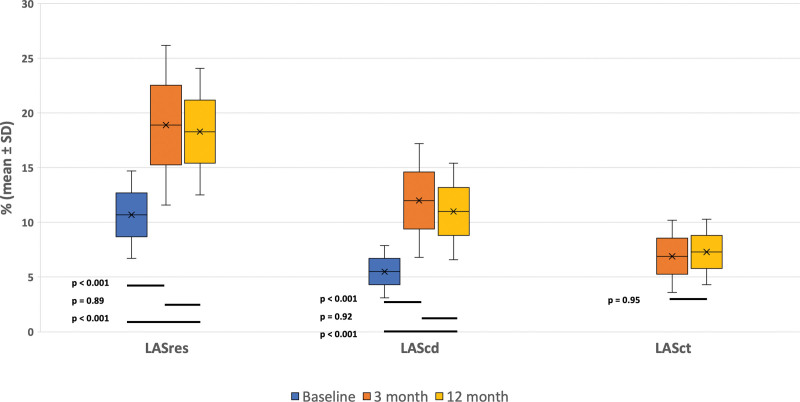
Changes in left atrial reservoir (LASres), conduit (LAScd), and contractile (LASct) function from baseline to 12 months in the total study population.

Compared with baseline, there were significant reductions in LAmax (48.8±13.8 versus 43.7±12.2 mL/m^2^; *P*=0.01) and LAmin (39.1±13.4 versus 30.6±11.7 mL/m^2^; *P*<0.001) with increases in LAEF (20.3±9.5% versus 30.9±10.9%; *P*<0.001) at 3 months following ablation, and these alterations were maintained at 12 months (Figure 3). A reduction in LA reverse remodeling was observed (−7.3±26.9%) at 3 months following ablation. There was an increase in the LA sphericity index from baseline to 12 months (70.6±11.4% versus 74.7±10.6%; *P*=0.001) due to greater shortening of the LA anteroposterior length relative to transverse diameter, as shown in Figure 4. The LA strain data showed significant improvements in the absolute values of LASres (10.8±4.0% versus 18.7±7.4%; *P*<0.001) and LAScd (5.6±2.4% versus 11.9±5.2%; *P*<0.001) at 3 months, which were maintained at 12 months (11.9±5.2% versus 11.0±4.5; *P*=0.92) as shown in Figure 4. There was also a recovery of LASct following ablation at 3 and 12 months (17.2±11.3% versus 17.1±10.5%; *P*=0.95), as shown in Figure 4.

LV diastolic function was assessed using LV e’ and LV E/e’ ratio at baseline as the patients were in AF. LV e’ was normal at baseline and 3 months following ablation (9.8±2.4 versus 8.3±2.0 cm/s; *P*<0.001) and maintained at 12 months (8.3±2.0 versus 8.1±2.2 cm/s; *P*=0.54). LV E/e’ was also normal at baseline (9.5±3.2) and maintained at 3 months (9.5±3.7) and 12 months (10.1±4.7) following ablation.

### Comparison of Baseline Demographic Characteristics and LA Volumes Data Between the SR and AF Recurrence Groups

Thirty patients (36%) maintained SR throughout follow-up and AF recurred in 53 (64%) patients. The baseline demographic characteristics were similar between the 2 rhythm groups, as shown in Table 1.

There were no differences in LA sphericity index, LAmax, and Lap-wave volumes at baseline or follow-up, along with the extent of LA reverse remodeling (Table 2). Similarly, there was no significant difference in delta LAmax volume at 3 months between the 2 groups (−4.0±12.5 versus −5.8±11.7 mL/m^2^; *P*=0.51). There was a trend toward lower LAmin volume in the SR group compared with the AF recurrence group at 12 months (27.8±13.4 versus 32.6±12.1 mL/m^2^; *P*=0.06). Moreover, LAEF was significantly higher in the SR group at 3 months (36.3±10.6% versus 27.9±9.9%; *P*<0.001) and 12 months (37.2±9.9% versus 28±11.2%; *P*<0.001). This was also reflected by a higher delta LAEF in the SR compared to the AF recurrence group (15.7±10.3% versus 7.5±11.9%; *P*=0.002).

**Table 2.**
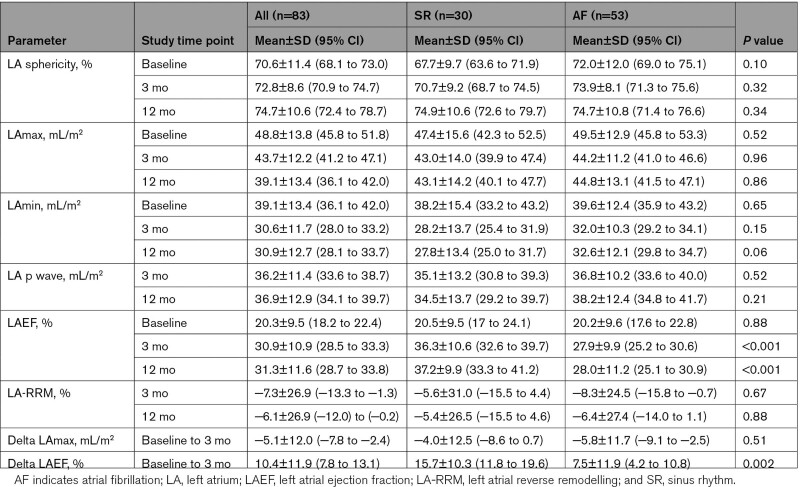
Comparison of Left Atrial Volumes Data Between the Sinus Rhythm and Atrial Fibrillation Recurrence Groups

### Comparison of LA Strain Data Between the SR and AF Recurrence Groups

LASres was significantly higher in the SR compared with the AF recurrence group at 3 months (22.6±8.5% versus 16.7±5.7%; *P*=0.001), and this was maintained at 12 months (20.8±6.1% versus 16.8±5.2%; *P*=0.003), see Table 3. Greater improvement in absolute delta LASres units between baseline and 3 months was seen in the group that maintained SR compared with those with AF recurrence (11.3±9.0% versus 6.2±4.9%; *P*=0.003). Similarly, LASct at 3 months was also greater in the SR compared with the AF recurrence group (9.2±3.4% versus 5.6±2.5%; *P*<0.001). By contrast, the increase in LAScd measurements at 3 and 12 months were similar in both groups.

**Table 3.**
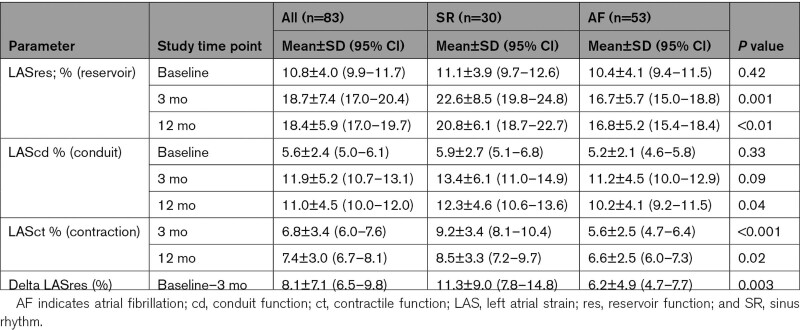
Comparison of Left Atrial Strain Data Between the Sinus Rhythm and Atrial Fibrillation Recurrence Groups

### Comparison of LV Diastolic Function Parameters Between the SR and AF Recurrence Groups

There was no significant change in E/e’ from baseline to 3 months in either the SR (8.7±3.1 versus 7.9±2.2; *P*=0.25) or AF recurrence group (9.9±3.2 versus 10.4±4.1; *P*=0.49). However, e’ velocities were significantly reduced from baseline to 3 months in the AF recurrence group (9.6±2.4 versus 7.9±1.9 cm/s; *P*<0.001) compared with the SR group (10.1±2.3 versus 9.1±2.1 cm/s; *P*=0.08).

As shown in Table 4, lower LV E/e’ (7.9±2.2 versus 10.4±4.1; *P*<0.001) and lower E/A ratios (1.5±0.50 versus 2.2±1.2; *P*<0.001) were observed at 3 months in the SR compared to the AF recurrence group. LV e’ at 3 months was higher in those who maintained SR compared with those who had AF recurrence (9.1±2.1 versus 7.9±1.9 cm/s; *P*=0.02).

**Table 4.**
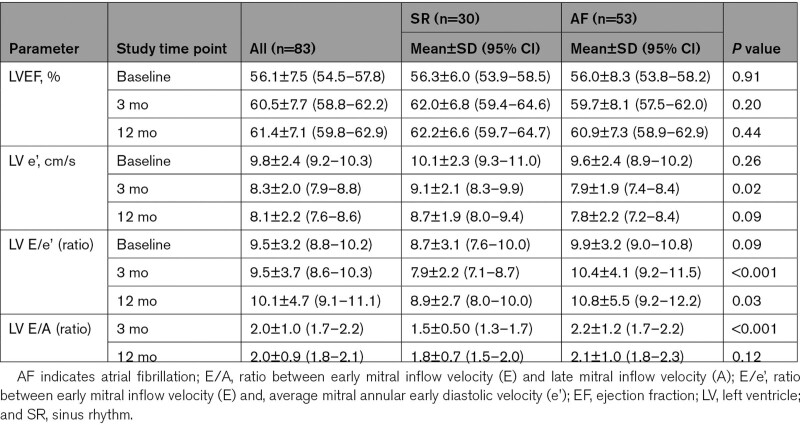
Comparison of Left Ventricular Systolic and Diastolic Function at Baseline and Follow-Up in the Sinus Rhythm vs Atrial Fibrillation Recurrence Groups

### Univariable and Multivariable Analysis for the Prediction of AF Recurrence

Univariable analysis of the baseline parameters showed that LA sphericity index and coronary artery disease were associated with a trend towards AF recurrence (Table 5). However, because it was present in only 10 patients, 13% of the sample, it was not included in the multivariable model.

**Table 5.**
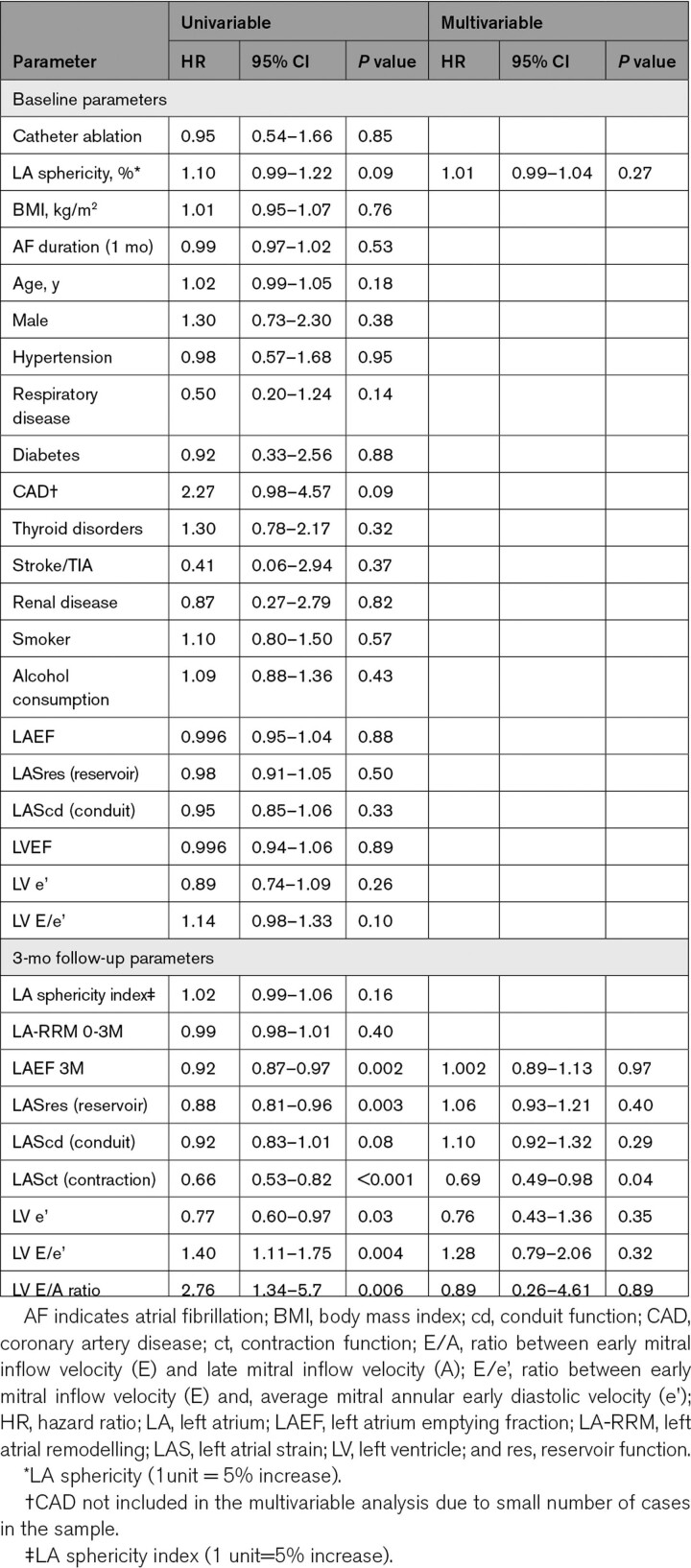
Univariable and Multivariable Cox Regression Analysis of Parameters Measured at Baseline and 3 mo After Ablation for the Prediction of Atrial Fibrillation Recurrence During Follow-Up

Univariable analysis of the 3-month follow-up data showed LAEF, LASres, LV e’ and LASct were inversely related to AF recurrence (Table 5). Higher LV E/e’ and E/A ratios at 3 months were associated with AF recurrence. Multivariable analysis showed reduced LASct is the only independent predictor of AF recurrence (hazard ratio, 0.69 [95% CI, 0.49–0.98]; *P*=0.04). Replacing the 3-month LAEF and LASres data in the multivariable model with delta LAEF and delta LASres did not affect this finding.

### Receiver Operating Characteristic Curve Analysis and Kaplan-Meier Survival Curve Analysis for LASct

**Figure 5.**
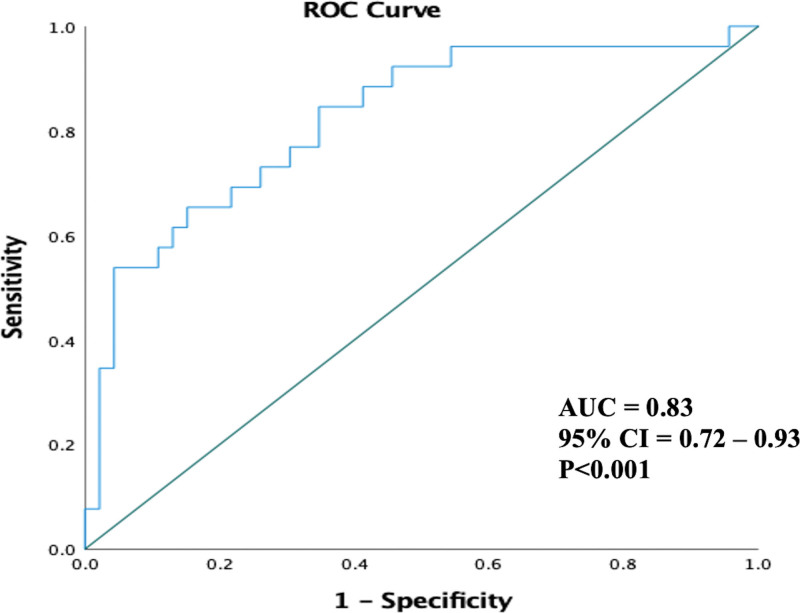
Receiver operating characteristic (ROC) curve of left atrial contractile strain at 3 months for predicting atrial fibrillation recurrence.

**Figure 6.**
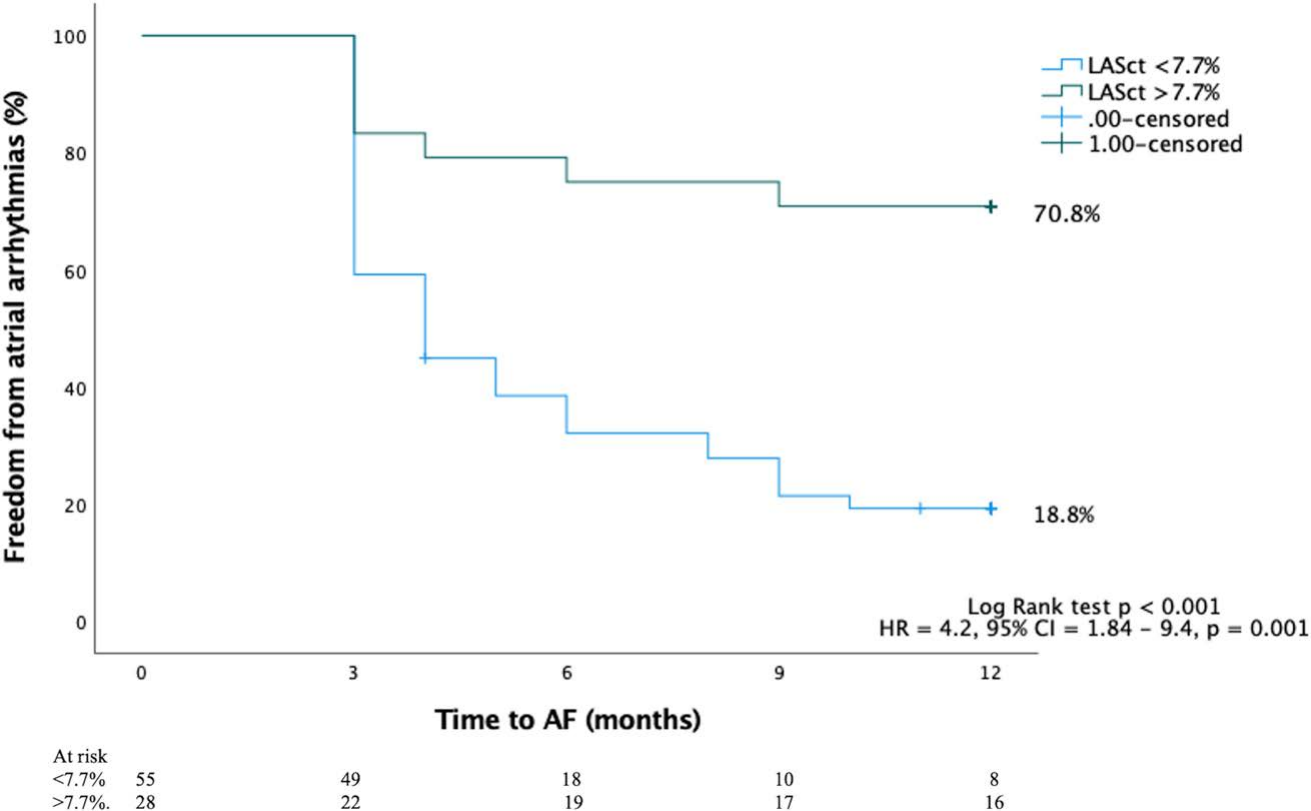
**Kaplan-Meier survival curves based on a left atrial strain contraction (LASct) cutoff value of 7.7% measured at 3-month follow-up.** AF indicates atrial fibrillation; and HR, hazard ratio.

Receiver operating characteristic curve analysis for LASct at 3-month follow-up (Figure 5) showed strong predictive value for AF recurrence with an area under the curve of 0.83 (*P*<0.001) and an optimal cutoff value of 7.7% (sensitivity=62% and specificity=85%; positive predictive value=71%, negative predictive value=82%). As shown in Figure 6, a LASct value of >7.7% was associated with 70.8% freedom from AF, whereas a value of <7.7% was associated with 18.8% freedom from AF (hazard ratio, 4.2 [95% CI, 1.84–9.4]; *P*=0.001).

## Discussion

Our findings demonstrate beneficial structural and functional alterations of the LA following AF ablation manifest by a recovery in LASct and improvement in LASres and LAScd, leading to a reduction in LA volumes and an increase in LAEF. There was no significant change in the parameters of LV diastolic function following ablation. From the pathophysiological perspective, the primary benefit of AF ablation is the regulation of cardiac rhythm from AF to SR leading to the recovery of LASct. These changes augment LA emptying, thereby reducing LA volume at the end of LA contraction allowing greater scope for LA recoil during LA filling and thus improving reservoir function. The regulation of cardiac rhythm leads to a constant stroke volume through the LA, and the reduction in the LAmin volume, facilitated by enhanced LA contraction, consequently leads to a reduction in LAmax volume. The LA volume reduction following ablation may also be related to scar-related contraction from ablation as seen in all our patients regardless of rhythm outcome, and the effects of underlying neurohormonal mechanisms.^25,26^

Approximately two-thirds of patients developed paroxysms of AF after the 3 months blanking period.^8^ Comparison of the baseline echocardiographic parameters between the two rhythm groups showed no difference in LA structure or function, and none of these parameters were predictive of AF recurrence. During the follow-up period, there were similar reductions in LAmax in both rhythm groups, but a trend toward lower LAmin in the SR group, leading to a significantly greater LAEF in this group compared with the AF recurrence group. Greater improvements in LASres and LASct, along with improved LV diastolic filling parameters were noted in the SR compared with the AF recurrence group at 3 months. Patients with AF recurrence had higher E/A and E/e’ ratios and lower e’ than the SR group at 3 months following ablation suggesting higher LV filling pressures in the AF recurrence group. Previous studies in paroxysmal AF and persistent AF have shown LV diastolic function parameters to predict AF recurrence following ablation,^27,28^ but this was not the case in our LSPAF sample. Notably, LA function is responsible for adequate LV filling, and it has been shown that the assessment of LA function by strain imaging may be more sensitive than measuring conventional LV filling parameters in determining the presence of LV diastolic dysfunction.^29,30^ In our study, LA strain at baseline was low and not predictive of AF recurrence following ablation. However, at the 3-month time point, the only independent predictor of AF recurrence was reduced LASct, indicating that the lower the extent of LA myocardial shortening during atrial systole, the greater the likelihood of AF recurrence.

As with LV myocardial physiology, the Frank-Starling mechanism also applies to the LA myocardium, such that as LA diameter and reservoir function increase, LA contractile function also increases, thereby maintaining stroke volume. However, in the presence of severe LA dilatation when the optimal pressure-volume relationship is exceeded, LA reservoir and contractile function might also decrease. It is well recognized that the longer the duration of AF, the more advanced the pathophysiological alterations leading to increasing LA dilatation and dysfunction.^7,31^ Using various imaging modalities to assess LA structure, larger LA volume has been shown to be a strong predictor of AF recurrence in patients with predominantly paroxysmal AF undergoing catheter ablation.^2^ In a study using 3D echocardiography, in patients with paroxysmal AF, an optimal LA volume threshold of 44.1 mL/m^2^ was predictive of AF recurrence. Although LA volumes derived from 3D echocardiography tend to be higher than 2D measurements, this was lower than the mean LA max of 48.8 mL/m^2^ in our study sample, reflecting the longer duration of persistent AF and more adverse LA remodeling in our patients.^7,26^ Liu et al^7^ compared paroxysmal, persistent AF, and LSPAF showing a reduction in LAEF, LASres, and LASct while seeing an increase in LAmax as the duration of AF increased. Regarding LA function, it has been shown in patients with paroxysmal AF undergoing catheter ablation, that the use of 2D strain can detect functional alterations linked to the recurrence of AF even when LA volumes are normal.^30^ A meta-analysis of 8 studies using 2D speckle tracking echocardiography showed that LA strain was the strongest baseline echocardiographic predictor of AF recurrence in patients undergoing catheter ablation.^32^ However, most patients had paroxysmal AF and none of the studies were in patients with persistent AF alone. This is reflected by a substantially lower mean LA volume index of 34 mL/m^2^ compared with our LSPAF patients. Moreover, the LA strain measurements predominantly reflected reservoir function, while reservoir and contractile strain values were not presented as 2 separate entities. Similarly, in studies including persistent AF and LSPAF patients, an LA strain of <10% has shown to be a strong predictor of AF recurrence.^6,33^ By contrast, our study, including only patients with LSPAF, was not able to identify a baseline echocardiographic predictor of AF recurrence to guide the need for catheter ablation. This most likely reflects the advanced adverse LA remodeling, blunting any discriminatory power of the echocardiographic data to predict AF recurrence. A study by Hanaki et al. recruited patients with persistent AF but cardioverted them electrically before final recruitment and before ablation.^33^ Patients who failed to maintain SR before ablation had worse LA strain reservoir function compared to the patients who remained in SR before ablation. In our sample, we exclusively recruited patients with continuous AF for more than 1 year, including those with failed cardioversion, representing a more advanced disease state. We also measured all 3 components of the LA function, not just the reservoir. The baseline LA volume in our study sample was nearly 2-fold higher than a healthy population of similar age,^34^ and although improved after AF ablation, the LA strain values remained substantially lower than those observed in a normal healthy population or milder categories of AF.^35^ Wen et al reported LA contraction as a predictor of AF recurrence when measured on day-1 post-ablation. Subsequent measurements at 3 months were not predictive of AF recurrence. It is unlikely that LA contraction measured so close to the ablation is clinically relevant because it takes time for the tissues to heal and heart rhythm to stabilize.^36^ A recent meta-analysis of a healthy population found the mean LASres was 39.4%, LAScd was 23%, and LASct was 17.4%.^35^ In our study sample, LASres, LAScd and LASct improved following ablation to less than half of these values. Nevertheless, our study demonstrates the pathophysiological importance of LA contractile strain in maintaining SR and predicting AF recurrence following AF ablation. Even in our LSPAF sample, low LA contractile strain following SR restoration could identify those at higher risk of AF recurrence. This finding reaffirms the view that assessing LA function rather than volume can more accurately measure the impact of LA substrate remodeling resulting from neurohormonal conditions present in LSPAF.^9,26^

Cardiac magnetic resonance imaging and histopathology have shown a strong inverse correlation of LA fibrosis with LA strain in patients with persistent AF.^37–40^ In addition, a significant link exists between the severity of LA fibrosis analyzed by cardiac magnetic resonance and AF recurrence after catheter ablation.^41–43^ Echo and cardiac magnetic resonance–based speckle tracking to detect LASct and LASres significantly correlates to low LA voltage areas during ablation.^44,45^ Greater improvements in LASct and LASres in the SR group we observed are also likely to reflect the less fibrotic change and better LA compliance than in those with AF recurrence.

### Limitations

The main limitation of this study was the relatively small sample size, in part determined by the availability of good quality echocardiographic images for all relevant time points and less so the presence of sinus rhythm during follow-up to measure contractile function. High-quality images were essential for LA strain measurements due to respiratory motion artifact and unfavourable body habitus. Consequently, the cutoff value of LA strain found to predict AF recurrence in LSPAF proposed in this study may not be robust and larger studies are required to explore this further. Our proposed cutoff value only represents a LSPAF cohort, as studies of paroxysmal AF show better contractile function. Three-dimensional echocardiography may have provided more accurate LA volume measurements but is technically challenging, requires very high-quality images, and would almost certainly have led to further exclusions from our study. It is important to acknowledge that the measurement of LA volume is a part of the minimum dataset for transthoracic echocardiography in routine clinical practice, whereas assessment of LA strain remains a research tool with a need for further standardization of techniques and improved reproducibility.^24^

### Conclusions

LA function in LSPAF improves after AF ablation as shown by a reduction in LA volumes, improved LAEF, better reservoir function and recovery of atrial contraction. Reduced LASct following the blanking period is the only independent predictor of AF recurrence. Further studies are needed to review ablation outcomes based not only on LA structure but also on LA function, particularly LASct, which might lead to improved ablation success.

## Article Information

### Sources of Funding

The funding of the CASA AF Trial (Catheter Ablation vs. Thoracoscopic Surgical Ablation in Long Standing Persistent Atrial Fibrillation) was through the Efficacy and Mechanism Evaluation (EME) Program, a Medical Research Council (MRC) and National Institute for Health Research (NIHR) partnership (grant number 12/127/ 127). The views expressed in this publication are those of the author(s) and not necessarily those of the MRC, NIHR, or the Department of Health and Social Care.

### Disclosures

Dr Khan received presentation fees from CHRS and NIHR grant (EME 12/127/127); Dr Haldar received a grant from NIHR (EME 12/127/127), Abbott, presentation fees from Alivecor and Zurich Heart House; Dr Bahrami received honoraria fees from Medtronic and meeting support from Edwards Lifesciences; Dr De Souza received presentation/support fees from Medtronic and Atricure. Dr De Souza is serving as BISICS President; Dr Markides received educational grants, consultancy fees, and honoraria from Biosense Webster; Dr Jones receives institutional research grants from Biosense Webster, Boston Scientific, and Medtronic; the rest of the authors disclose no conflicts of interest.

### Supplemental Material

Tables S1–S3

## Supplementary Material


