# Supplementary material for: Left Atrial Function Predicts Atrial Arrhythmia Recurrence Following Ablation of Long-Standing Persistent Atrial Fibrillation
Source: Circ Cardiovasc Imaging. 2023 Jun 8;16(6):e015352. doi: 10.1161/CIRCIMAGING.123.015352 (PMC10281195; doi:10.1161/CIRCIMAGING.123.015352)
Supplement: Supplementary file 1 [file hci-16-e015352-s001.pdf]

Supplementary Tables and Figures

SUPPLEMENTARY TABLES

Table S1: Echocardiographic measurements and equations for LA volume dataset.

LA volume max (LA $_{max}$)	0.85 x $\frac{(LA\ 4ch\ \max\ area\ cm2\ x\ LA\ 2ch\ \max\ area\ cm2)}{LA\ 4ch\ \max\ LA\ AP\ length\ cm}$
LA volume min (LA $_{min}$)	0.85 x $\frac{(LA\ 4ch\ \min\ area\ cm2\ x\ LA\ 2ch\ \min\ area\ cm2)}{LA\ 4ch\ \min\ LA\ AP\ length\ cm}$
LA volume at p wave (LA $_{P\ wave}$)	0.85 x $\frac{(LA\ 4ch\ \text{p wave}\ area\ cm2\ x\ LA\ 2ch\ \text{p wave}\ area\ cm2)}{LA\ 4ch\ \text{p wave}\ LA\ AP\ length\ cm}$
LA emptying fraction (LAEF%)	$\left[\frac{(LA\ volume\ max -\ LA\ volume\ min)}{LA\ volume\ max}\right]\text{x}100$
LA Sphericity (%)	$\left[\frac{\text{LA transverse diameter}}{LA\ anteroposterior\ length}\right]\text{x}100$
Reverse remodelling	$\frac{LAmax\ 3\ month - LAmax\ baseline}{LAmax\ baseline}\ x\ 100$

Table S2: Comparison of baseline characteristics of 83 CASA AF sub-study participants versus the excluded patients.

**BASELINE DEMOGRAPHIC DATA**	**Included Patients (83)**	**SR (n=30)**	**AF (n=53)**	**P value SR vs AF**	**Excluded patients (n=32)**	**P valve (Included vs Excluded)**
Age, years (±SD)	63.6 ± 9.7 (61.5 – 65.8)	61.1 ± 9.1 (57.7 – 64.6)	64.9 ± 9.8 (62.3 – 67.6)	0.09	59.2 ± 13.5 (54.7 – 63.2)	0.10
Sex, n = male (%)	61 (73.5)	24 (80.0)	37 (69.8)	0.20	28 (87.5)	0.11
AF duration, months; mean (95%CI)	22.8 ± 11.6 (20.2 – 25.3)	24.1 ± 12.2 (19.4 – 28.7)	22.0 ± 11.3 (19.0 – 17.4)	0.45	29.4 ± 19.1 (24 – 34.3)	0.08
Body mass index, kg/m^2; mean (±SD)	30.5 ± 5.0 (29.4 – 31.6)	30.2 ± 4.8 (28.3 – 32)	30.7 ± 5.1 (29.3 -32.1)	0.61	29.7 ± 5.2 (28.0. – 31.5)	0.46
Smoking, n (%)	6 (7.2)	3 (10.0)	3 (5.7)	0.42	5 (15.6)	0.18
Hypertension, n (%)	43 (51.8)	14 (46.7)	29 (54.7)	0.65	11 (34.4)	0.09
Diabetes, n (%)	6 (7.2)	2 (6.7)	4 (7.5)	0.93	3 (9.3)	0.70
Alcohol, n (%)	43 (51.8)	13 (43.3)	30 (56.6)	0.78	14 (43.8)	0.44
Stroke/TIA, n (%)	3 (3.6)	2 (6.7)	1 (1.9)	0.24	1 (3.1)	0.90

Thyroid disorders, n (%)	5 (6.0)	1 (3.3)	4 (7.5)	0.47	2 (6.3)	0.96
Coronary artery disease, n (%)	11 (13.3)	1 (3.3)	10 (18.9)	0.05	2 (6.3)	0.29
Renal disease, n (%)	5 (6.0)	2 (6.7)	3 (5.7)	0.81	3 (9.3)	0.53
Respiratory disease, n (%)	13 (15.7)	8 (26.6)	5(9.4)	0.05	5 (15.6)	0.99
**DRUG THERAPY**						
Betablocker, n (%)	70 (84.3)	23 (76.7)	47 (88.7)	0.36	26 (81.3)	0.69
Digoxin, n (%)	20(24.1)	7 (23.3)	13 (24.5)	0.99	8 (25)	0.92
Ca channel blocker, n (%)	10(12.0)	6 (20.0)	4 (7.5)	0.08	3 (9.3)	0.69
ACEi/ARB, n (%)	42 (50.6)	14 (46.7)	28 (52.8)	0.82	13 (40.6)	0.38
Diuretic, n (%)	25 (30.1)	8 (26.7)	17 (32.1)	0.81	6 (18.8)	0.22
AADs, n (%)	10 (12.0)	2 (6.7)	8 (15.1)	0.29	3 (9.3)	0.69
Statin, n (%)	40 (48.2)	12 (40.0)	28 (52.8)	0.49	17 (53.1)	0.64

Table S3: Comparison of baseline characteristics of 83 CASA AF sub-study participants based on ablation strategy.

**DEMOGRAPHIC DATA**	**All** **(83)**	**CA** **(n=46)**	**SA** **(n=37)**	**P value**
Age, years mean ± SD, (95% CI)	63.6 ± 9.7 (61.5 – 65.8)	62.2 ± 10.1 (59.2 – 65.2)	65.5 ± 8.9 (62.5 – 68.4)	0.13
Sex, n = male (%)	61 (73.5)	34 (74.0)	27 (73.0)	0.99
AF duration, months; mean ± SD (95%CI)	23.1 ± 11.7 (20.5 – 25.6)	21.4 ± 10.8 (18.2 – 24.6)	25.1 ± 12.6 (20.9 – 29.3)	0.16
Body mass index, kg/m^2; mean ± SD (95% CI)	30.5 ± 5 (29.4 – 31.6)	30.9 ± 5.5 (29.3 – 32.6)	30 ± 4.3 (28.6 – 31.5)	0.42
Smoking, n (%)	6 (7.2)	3 (6.5)	3 (8.1)	0.46
Hypertension, n (%)	43 (51.8)	21 (45.6)	22 (59.4)	0.27
Diabetes, n (%)	6 (7.2)	4 (8.7)	2 (5.4)	0.57
Alcohol, n (%)	43 (51.8)	26 (56.5)	17 (45.9)	0.67
Stroke/TIA, n (%)	3 (3.6)	0	3 (8.1)	0.05
Thyroid disorders, n (%)	5 (6.0)	2 (4.4)	3 (8.1)	0.48
Coronary artery disease, n (%)	11 (13.3)	6 (13)	5 (13.5)	0.95
Renal disease, n (%)	5 (6.0)	4 (8.7)	1 (2.7)	0.26
Respiratory disease, n (%)	13 (15.7)	5 (10.8)	8 (21.6)	0.18

**DRUG THERAPY**				
Betablocker, n (%)	70 (84.3)	39 (84.7)	31 (83.8)	0.99
Digoxin, n (%)	20 (24.1)	14 (30.4)	6 (16.2)	0.20
Ca channel blocker, n (%)	10(12.0)	5 (10.9)	5 (13.5)	0.71
ACEi/ARB, n (%)	42 (50.6)	23 (50.0)	19 (51.4)	0.98
Diuretic, n (%)	25 (30.1)	11 (23.9)	14 (37.8)	0.23
AADs, n (%)	10 (12.0)	5 (10.9)	5 (13.5)	0.75
Statin, n (%)	40 (48.2)	19 (41.3)	21 (56.8)	0.19
